# Supplementary material for: Polygenic Susceptibility of Aortic Aneurysms Associates to the Diameter of the Aneurysm Sac: the Aneurysm-Express Biobank Cohort
Source: Sci Rep. 2019 Dec 27;9:19844. doi: 10.1038/s41598-019-56230-3 (PMC6934821; doi:10.1038/s41598-019-56230-3)
Supplement: Supplementary file 1 — Supplementary Material [file 41598_2019_56230_MOESM1_ESM.pdf]

Supplementary material

Manuscript title: Polygenic Susceptibility of Aortic Aneurysms Associates to the Diameter of the Aneurysm Sac: the Aneurysm-Express Biobank Cohort.

Authors: van Laarhoven CJHCM, van Setten J, van Herwaarden JA, Pasterkamp G, de Kleijn DPV, de Borst GJ, van der Laan SW.

Contents:

- Supplemental Figure 1
- Supplemental Figure 2
- Supplemental Table 1
- Supplemental Table 2
- Supplemental Table 3
- Supplemental Table 4
- Supplemental Table 5

Supplemental Figure 1

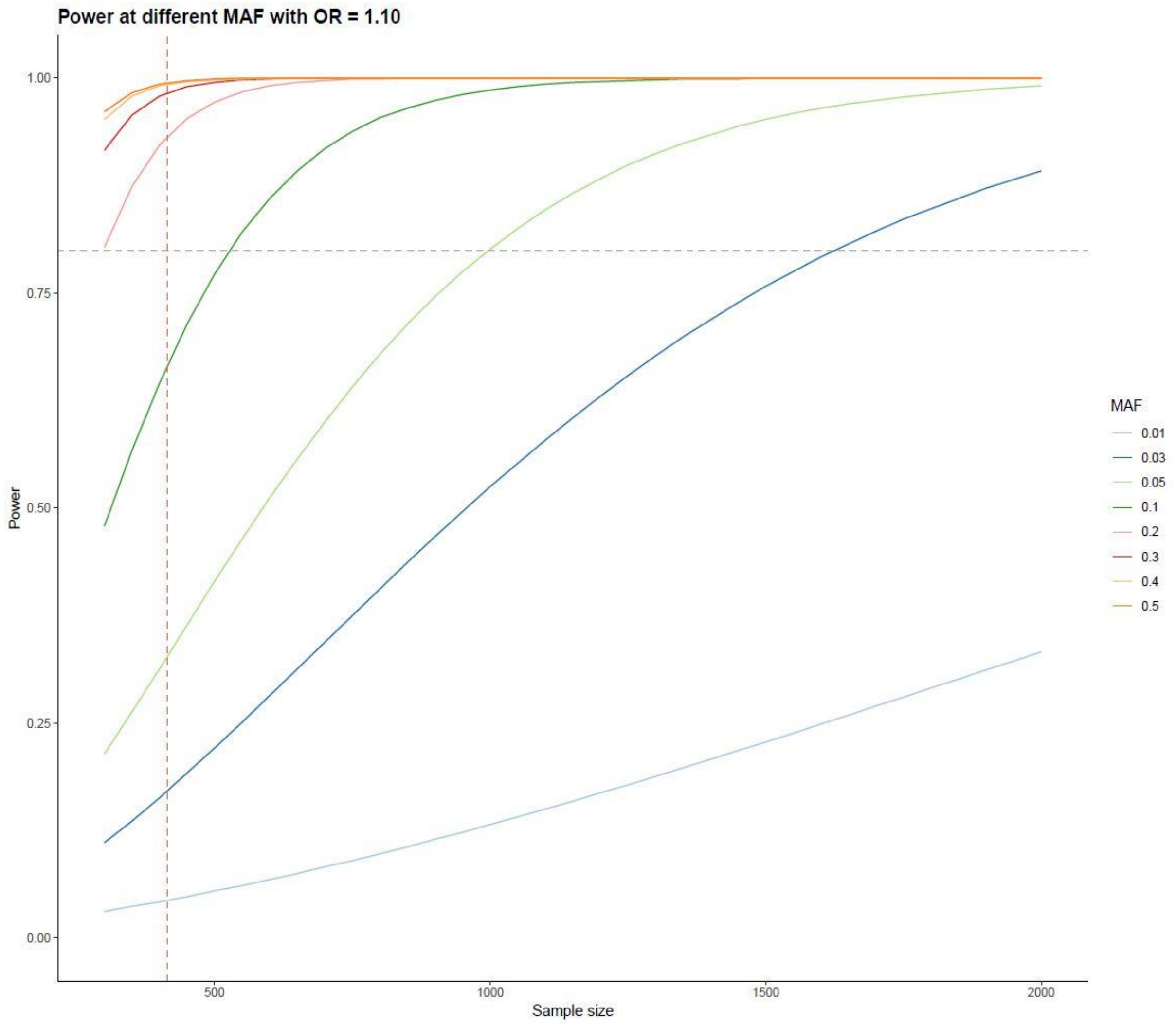

Supplemental Figure 1. Plot indicating study power estimates at different minor allele frequencies (MAF), with an average odds ratio (OR) of 1.10. Each solid colored line indicates a different MAF. Red dashed line indicates present study size of 415 samples, grey dashed line indicates a power estimate of ±80% for risk variants MAF < 0.2.

Supplemental Figure 2

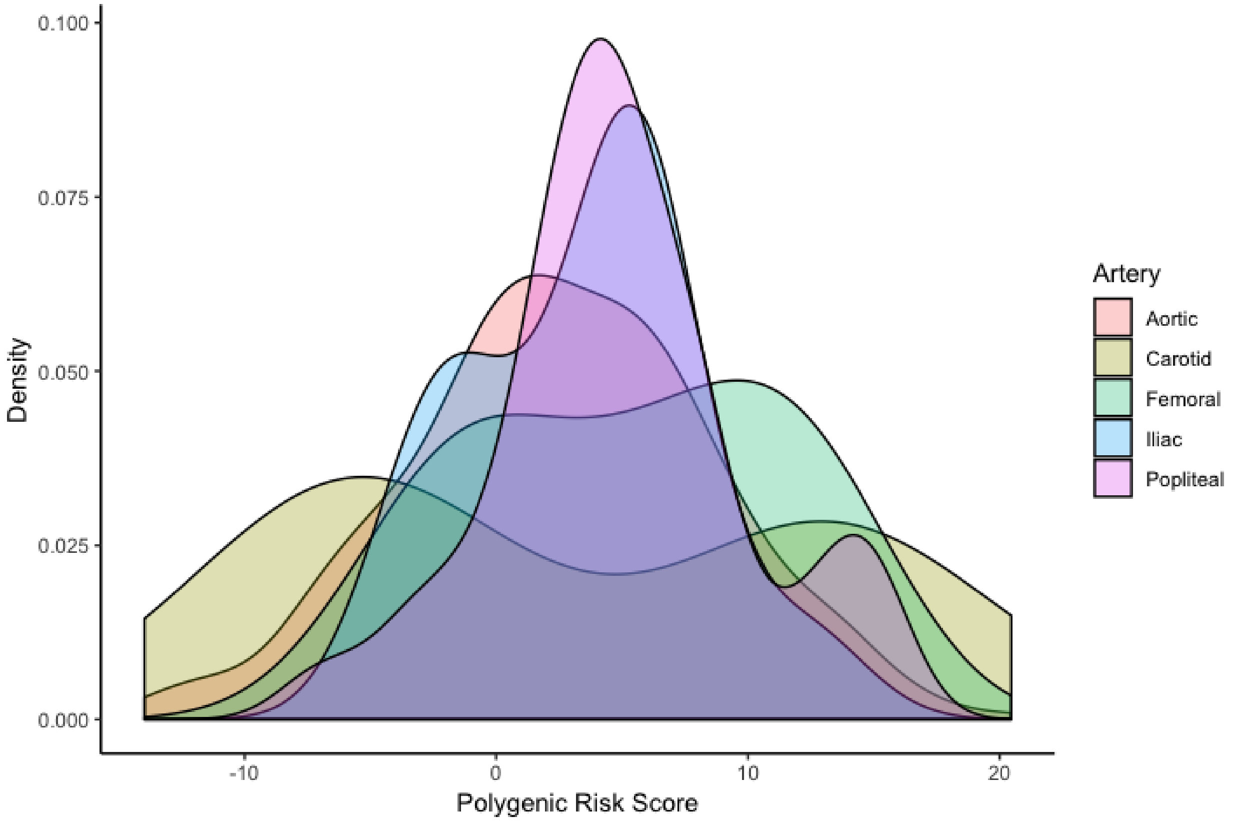

Supplemental Figure 2. Overlay density plot of polygenic risk score (PRS) for diameter per artery type. Distribution of PRS is comparable per artery type (*Kruskal-Wallis test p*-value = 0.135).

Supplemental Table 1. Polygenic models.

A baseline model was constructed correcting for age, sex, ancestral background using four principal components, smoking status, and maximum diameter of the aneurysm sac or artery type if applicable. p_{T}: the GWAS p-value threshold of association.

**Model**	**Includes**
GWAS	Baseline + GWAS hits p_T <5x10^{-8}
p < 5x10^{-6}	Baseline + all SNPs p_T <5x10^{-6}
p < 5x10^{-4}	Baseline + all SNPs p_T <5x10^{-4}
p < 0.001	Baseline + all SNPs p_T <0.001
p < 0.01	Baseline + all SNPs p_T <0.01
p < 0.05	Baseline + all SNPs p_T <0.05
p < 0.1	Baseline + all SNPs p_T <0.1
p < 0.2	Baseline + all SNPs p_T <0.2
p < 0.5	Baseline + all SNPs p_T <0.5

Supplemental Table 2. Detailed polygenic risk score (PRS) models derived from AAA GWAS [12] for selected clinical phenotypes.

p_T	R^2	β	SE	*p*-value	Number of SNPs
**Diameter of the aneurysm sac**					
$5x10^{-8}$	0.001	-1.932	2.143	0.368	1
$5x10^{-6}$	0.000	0.075	1.419	0.958	3
$5x10^{-4}$	0.001	0.371	0.431	0.390	24
0.001	0.004	0.493	0.299	0.100	40
0.01	0.018	0.394	0.116	0.001*	267
**0.01015**	**0.019**	**0.398**	**0.115**	**0.001***	**272**
0.05	0.004	0.095	0.061	0.120	1,008
0.1	0.004	0.075	0.046	0.105	1,634
0.2	0.004	0.054	0.036	0.133	2,494
0.5	0.004	0.045	0.029	0.125	4,086
**Artery type**					
p_T	R^2	β	SE	*p*-value	Number of SNPs
$5x10^{-8}$	0.005	-0.886	0.681	0.193	1
$5x10^{-6}$	0.003	-0.382	0.411	0.353	3
$5x10^{-4}$	0.000	0.055	0.138	0.688	24
0.001	0.004	0.110	0.096	0.252	39
**0.0022**	**0.007**	**0.099**	**0.063**	**0.114**	**88**
0.01	0.007	0.056	0.038	0.140	267
0.05	0.030	0.020	0.020	0.321	1,008
0.1	0.001	0.006	0.014	0.652	1,632
0.2	0.000	0.000	0.012	0.989	2,493
0.5	0.000	-0.001	0.009	0.950	4,086
**Symptom status**					
p_T	R^2	β	SE	*p*-value	Number of SNPs
$5x10^{-8}$	0.000	0.032	0.342	0.925	1
$5x10^{-6}$	0.000	0.023	0.226	0.921	3
$5x10^{-4}$	0.003	0.064	0.069	0.350	24
0.001	0.001	0.025	0.049	0.604	39
0.01	0.002	0.013	0.019	0.491	270
0.05	0.002	0.007	0.010	0.483	1,009
**0.0637**	**0.007**	**0.012**	**0.009**	**0.173**	**1,213**
0.1	0.000	0.002	0.007	0.768	1,632
0.2	0.001	0.002	0.006	0.692	2,494
0.5	0.000	0.001	0.005	0.818	4,086

Bold indicates best fitted model, * indicates $p < 0.05$.

Abbrevations: p_T = *p*-value threshold. β = beta coefficient. SE = standard error, SNPs = single nucleotide polymorphisms.

Supplemental Table 3. Detailed polygenic risk score (PRS) models derived from attention deficit hyperactivity disorder GWAS [39] for selected clinical phenotypes.

Diameter of the aneurysm sac

p_T	R^2	β	SE	*p*-value	Number of SNPs
$5x10^{-8}$	0.003	-0.973	0.714	0.174	10
**$5x10^{-6}$**	**0.004**	**-1.105**	**0.703**	**0.117**	**77**
$5x10^{-4}$	0.000	-0.256	0.710	0.719	1,179
0.001	0.000	0.306	0.716	0.670	1,858
0.01	0.002	-0.702	0.727	0.335	8,155
0.05	0.002	-0.907	0.745	0.224	20,395
0.1	0.001	-0.494	0.745	0.507	28,657
0.2	0.000	-0.298	0.741	0.688	39,167
0.5	0.000	-0.409	0.741	0.581	55,850

Artery type

p_T	R^2	β	SE	*p*-value	Number of SNPs
$5x10^{-8}$	0.000	0.086	0.230	0.707	10
**$5x10^{-6}$**	**0.010**	**-0.424**	**0.234**	**0.070**	**77**
$5x10^{-4}$	0.001	-0.168	0.240	0.486	1,179
0.001	0.000	-0.045	0.239	0.851	1,858
0.01	0.001	-0.115	0.235	0.625	8,155
0.05	0.003	-0.254	0.239	0.289	20,395
0.1	0.000	-0.006	0.226	0.979	28,657
0.2	0.000	-0.008	0.232	0.972	39,167
0.5	0.000	-0.026	0.234	0.913	55,850

Symptom status

p_T	R^2	β	SE	*p*-value	Number of SNPs
$5x10^{-8}$	0.000	0.028	0.115	0.809	10
**$5x10^{-6}$**	**0.010**	**-0.184**	**0.114**	**0.106**	**77**
$5x10^{-4}$	0.001	-0.070	0.113	0.533	1,179
0.001	0.002	-0.081	0.114	0.481	1,858
0.01	0.000	-0.038	0.116	0.740	8,155
0.05	0.000	-0.032	0.119	0.791	20,395
0.1	0.001	-0.071	0.120	0.553	28,657
0.2	0.001	-0.074	0.119	0.531	39,167
0.5	0.000	-0.005	0.118	0.969	55,850

Bold indicates best fitted model, * indicates $p < 0.05$.
Abbrevations: p_T = *p*-value threshold. β = beta coefficient. SE = standard error, SNPs = single nucleotide polymorphisms.

Supplemental Table 4

Supplemental Table 4.
Clinical characteristics of the upper 20th percentile and lower 80th percentile of PRS.

	Lower 80th of PRS n = 332		Upper 20th of PRS n = 83		*p*
**Male gender**	285	(81%)	68	(19%)	.371 [a]
**Age at surgery,** median (range)	69.5	(46-89)	71.0	(28-87)	.460 [b]
**In-hospital days,** median (range)	9	(1-81)	10	(1-65)	.216 [b]
**Any in-hospital reintervention post-operatively**	37	(13%)	9	(12%)	.747 [a]
**In-hospital death**	16	(6%)	3	(4%)	.576 [a]
**Aneurysm**					
Aortic	281	(81%)	69	(19%)	.311 [a]
Iliac	8	(62%)	5	(39%)	
Femoral	7	(87%)	1	(13%)	
Popliteal	28	(80%)	7	(20%)	
Carotid	8	(89%)	1	(11%)	
**IL-6*,** mean±sd	4.0	± 1.8	3.7	± 2.3	.321 [c]
**IL-8***	4.6	± 1.5	4.1	± 1.6	.051 [c]
**MCP-1***	1.9	± 2.1	1.8	± 2.0	.782 [c]
**OPG***	8.3	± 1.0	8.3	± 1.0	.964 [c]

Abbreviations: PRS = polygenic risk score, IL = interleukin, sd = standard deviation, MCP = monocyte chemotactic protein, OPG = osteoprotegerin. * natural log transformed, all inflammatory markers are extracted from the tissue sample, analyzed by Luminex and corrected for inter assay variability with pooled and blank sample.
[a] Chi-squared test, [b] Mann-Whitney U test, [c] Student's T-test.

Supplemental Table 5

Supplemental Table 5.
AAA associated SNPs reported by GWAS and the association results for artery type and symptom status.

**Reported by literature**								**This study**							
								Artery type				*Symptom status*			
**SNP**	**Chr**	**BP**	**Near(est) gene(s)**	**Alleles**	**EAF**	**β [a]**	***p***	**EAF**	**β**	**SE**	***p***	**EAF**	**β**	**SE**	***p***
rs602633	1	109821511	*PSRC1-CELSR2-SORT1*	T* - G	0.199	-0.129	6.58×10^{-9}	0.208	0.034	0.306	0.911	0.208	-0.694	0.192	0.132
rs4129267	1	154426264	*IL6R*	T* - C	0.370	-0.132	4.76×10^{-13}	0.355	0.173	0.270	0.522	0.361	0.564	0.171	0.133
rs1795061	1	214409280	*SMYD2*	T* - C	0.337	0.123	8.80×10^{-11}	0.307	0.149	0.276	0.591	0.315	-0.550	0.165	0.125
rs10757274	9	22096055	*CDKN2BAS 1/ANRIL*	A* - G	0.462	-0.216	1.54×10^{-33}	0.504	-0.015	0.263	0.955	0.506	-0.377	0.160	0.276
rs10985349	9	124425243	*DAB2IP*	T* - C	0.195	0.158	2.40×10^{-11}	0.200	-0.015	0.339	0.964	0.204	0.149	0.200	0.711
rs1466535	12	57534470	*LRP1*	G* - A	0.679	0.199[b]	$9.99 \text{X} 10^{-7}$	0.655	0.039	0.267	0.885	0.655	-0.255	0.166	0.124
rs9316871	13	22861921	*LINC00540*	A* - G	0.201	-0.136	4.75×10^{-10}	0.796	0.176	0.348	0.613	0.794	0.037	0.197	0.930
rs6511720	19	11202306	*LDLR*	T* - G	0.096	-0.218	7.90×10^{-14}	0.094	0.306	0.469	0.514	0.098	0.069	0.273	0.902
rs3827066	20	44586023	*PCIF1-ZNF335-MMP9*	T* - C	0.179	0.201	2.13×10^{-17}	0.167	0.190	0.345	0.583	0.176	-0.879	0.207	0.048
rs2836411	21	39819830	*ERG*	T* - C	0.369	0.107	5.80×10^{-9}	0.361	0.433	0.311	0.164	0.364	-0.500	0.194	0.224

Abbreviations: AAA = abdominal aortic aneurysm, SNP = single nucleotide polymorphism, Chr = chromosome, BP = base pair, EAF = effect allele frequency, β = beta-coefficient, SE = standard error.
* Effect allele, [a] β converted from combined odds ratio's (discovery and validation phase) of summary statistics of *Jones et al.(2017)* [12].
[b] β converted from discovery phase of summary statistics of *Bown et al. (2011)* [7].
